# Development of a competency-based formative progress test with student-generated MCQs: Results from a multi-centre pilot study

**DOI:** 10.3205/zma000988

**Published:** 2015-10-15

**Authors:** Stefan Wagener, Andreas Möltner, Sevgi Tımbıl, Maryna Gornostayeva, Jobst-Hendrik Schultz, Peter Brüstle, Daniela Mohr, Anna Vander Beken, Julian Better, Martin Fries, Marc Gottschalk, Janine Günther, Laura Herrmann, Christian Kreisel, Tobias Moczko, Claudius Illg, Adam Jassowicz, Andreas Müller, Moritz Niesert, Felix Strübing, Jana Jünger

**Affiliations:** 1 University of Heidelberg, Faculty of Medicine, Heidelberg, Germany; 2 University of Heidelberg, Center of Excellence for Assessment in Medicine - Baden-Wuerttemberg, Heidelberg, Germany; 3 Albert-Ludwigs-University Freiburg, Freiburg Competence Center for Evaluation of Teaching in Medicine - Baden-Wuerttemberg, Freiburg, Germany; 4 University of Tübingen, Faculty of Medicine, Tübingen, Germany; 5 University of Ulm, Faculty of Medicine, Ulm, Germany; 6 University of Marburg, Faculty of Medicine, Marburg, Germany; 7 University of Magdeburg, Faculty of Medicine, Magdeburg, Germany; 8 University of Freiburg, Faculty of Medicine, Freiburg, Germany; 9 University of Witten/Herdecke, Faculty of Medicine, Witten, Germany

**Keywords:** progress test, competency-based, medical students, medical education, multiple-choice questions

## Abstract

**Introduction:** Progress tests provide students feedback on their level of proficiency over the course of their medical studies. Peer-assisted learning and competency-based education have become increasingly important in medical education. Although progress tests have been proven to be useful as a longitudinal feedback instrument, there are currently no progress tests that have been created in cooperation with students or that focus on competency in medical education.

In this study, we investigated the extent to which students can be included in the development of a progress test and demonstrated that aspects of knowledge related to competency can be represented on a competency-based progress test.

**Methods: **A two-dimensional blueprint for 144 multiple-choice questions (MCQs) covering groups of medical subjects and groups of competency areas was generated by three expert groups for developing the competency-based progress test. A total of 31 students from seven medical schools in Germany actively participated in this exercise. After completing an intensive and comprehensive training programme, the students generated and reviewed the test questions for the competency-based progress test using a separate platform of the ItemManagementSystem (IMS). This test was administered as a formative test to 469 students in a pilot study in November 2013 at eight medical schools in Germany. The scores were analysed for the overall test and differentiated according to the subject groups and competency areas.

**Results: **A pool of more than 200 MCQs was compiled by the students for pilot use, of which 118 student-generated MCQs were used in the progress test. University instructors supplemented this pool with 26 MCQs, which primarily addressed the area of scientific skills. The post-review showed that student-generated MCQs were of high quality with regard to test statistic criteria and content. Overall, the progress test displayed a very high reliability. When the academic years were compared, the progress test mapped out over the course of study not only by the overall test but also in terms of the subject groups and competency areas.

**Outlook:** Further development in cooperation with students will be continued. Focus will be on compiling additional questions and test formats that can represent competency at a higher skill level, such as key feature questions, situational judgement test questions and OSCE. In addition, the feedback formats will be successively expanded. The intention is also to offer the formative competency-based progress test online.

## Zusammenfassung

**Einleitung: **Progresstests geben Studierenden Feedback über ihren Wissenszuwachs im Verlauf ihres Studiums. In der medizinischen Ausbildung haben Peer-assisted-learning und kompetenzbasierte Ausbildung zunehmend Bedeutung erlangt. Während Progresstests sich als longitudinales Feedback-Instrument bewährt haben, gibt es bisher keine Progresstests, die in Zusammenarbeit mit Studierenden erstellt wurden und die die Kompetenzorientierung der medizinischen Ausbildung aufgreifen.

In dieser Studie soll zum einen untersucht werden, inwieweit Studierende in die Prüfungsentwicklung eines Progresstests eingebunden werden können und zum anderen soll gezeigt werden, dass sich die Wissensaspekte von Kompetenzen in einem kompetenzorientierten Progresstest abbilden lassen.

**Methoden:** Für den kompetenzorientierten Progresstest wurde ein zweidimensionaler „Blueprint“ für 144 MC-Fragen mit gruppierten medizinischen Fächern und gruppierten Kompetenzbereichen von drei Expertengruppen erstellt. An der Entwicklung des kompetenzorientierten Progresstests haben sich 31 Studierende aus sieben medizinischen Fakultäten in Deutschland aktiv beteiligt. Nach einem intensiven und umfassenden Schulungsprogramm erstellen und reviewten die Studierenden unter Nutzung einer eigenständigen Plattform des ItemManagementSystems die Prüfungsfragen des kompetenzorientierten Progresstests. Der kompetenzorientierte Progresstest wurde im November 2013 an acht medizinischen Fakultäten in Deutschland als formativer Test im Rahmen einer Pilotstudie durchgeführt, an dem 469 Studierende teilnahmen. Die erzielten Punktwerte wurden für den Gesamttest sowie in differenzierter Form für die „Fächergruppen“ und „Kompetenzbereiche“ analysiert.

**Ergebnisse: **Für den Piloteinsatz im Jahr 2013 wurde von den Studierenden ein MC-Fragenpool von über 200 MC-Fragen entwickelt. 118 MC-Fragen von Studierenden wurden im Progresstest verwendet. 26 MC-Fragen wurden von Dozenten insbesondere im Bereich Wissenschaftskompetenz ergänzt. Es zeigte sich, dass die MC-Fragen von Studierenden hinsichtlich teststatistischer Kriterien und inhaltlicher Prüfung im Postreview von hoher Qualität waren. Der Progresstest weist insgesamt eine sehr hohe Reliabilität auf. Im Vergleich der Studienjahre bildete sich der Progress im Verlauf des Studiums sowohl in der Gesamtbetrachtung des Tests als auch hinsichtlich der „Fächergruppen“ und „Kompetenzbereiche“ ab.

**Ausblick: **Die Weiterentwicklung in Kooperation mit Studierenden wird fortgesetzt. Im Fokus steht dabei die Erweiterung auf zusätzliche Fragen- und Prüfungsformate, die Kompetenzorientierung auf einem höheren Kompetenzlevel abbilden können, wie z.B. Key Feature-Fragen, Situational-Judgement-Test-Fragen und OSCE. Des Weiteren werden die Feedbackformate sukzessive erweitert. Auch ist geplant, den formativen kompetenzorientierten Progresstest webbasiert anzubieten.

## Introduction

The progress test is internationally recognized and used as a method for assessing learning progress over the course of university study [1], and it is also used in Germany by various university faculties [2]. Progress tests primarily fulfil two functions: they provide continual feedback to students about their level of knowledge during medical study and they also make it possible for universities to monitor curricula and cohort progress and to compare different curricula [3]. One special aspect of the progress test is its design, which is independent of a specific curriculum and allows for inter-university cooperation in test design, generation and administration [4], [5].

The active involvement of students in teaching has been anchored for many years in several educational systems. Using peer-assisted learning (PAL) as a teaching mode, experienced students (seniors) are usually made to instruct younger students (juniors) [6], [7], [8]. Studies on training practical clinical skills have shown that trained tutors are just as able as instructors to impart procedural clinical techniques, such as physical examinations, communicative skills, surgical skills and the clinical skills required in hospital wards [9], [10], [11], [12], [13]. It has also been shown that communicative skills integrated with practical clinical skills can be effectively taught by student tutors during the pre-clinical phase [14]. Emphasis is placed on the idea that more advanced students, as peers, are better able to put themselves into the position of the younger students who do not (yet) know very much [15] and that instruction by peers is found to be more comfortable [16]. Frameworks now show how PAL can be implemented in medical education [17], [18].

In contrast to teaching, students are much less involved in assessments (peer assessment). In a few studies, some involving medical education, students were shown to participate in the drafting of MCQs [19], [20], which proved valuable for generating questions for a test question bank [21], [22]. The influence of writing MCQs on learning strategies and behaviour has also been addressed [20], [21], [23]. Analysis of the quality of student-generated MCQs shows that students are able to create good quality MCQs [24], with indications that student-generated questions can be of the same level of quality as those written by instructors [22]. To date, we are unaware of studies in which students have developed tests completely on their own.

The positive experiences with PAL and peer assessment make the involvement of students both sensible and promising:

1. The students’ point of view on a formative and voluntary progress test is important for the design and use of a progress test. Students are able to provide important information regarding needs assessment, relevance and motivation to participate.
2. By involving students in the process, steps can be taken towards creating a positive testing culture. Students can share their needs in terms of the overall concept of the progress test and gain the opportunity to participate in the creation of a test, whose feedback is helpful for their academic development.
3. Student participation can provide insight on the extent to which students are able to not only generate a progress test in terms of content and organisation but also advance, implement and even develop it further in future.
4. By involving students in the process, it is possible to explore the extent to which a progress test can also be organized by and administered at multiple universities.

In Germany, the international development of competency-based education [25], [26] has led to the drafting of a national catalogue of competency-based learning objectives for undergraduate medical and dental education (NKLM/NKLZ) [http://www.nklm.de, last verified on 14 December 2014]. The objective of the NKLM/NKLZ is to describe the competencies that may be expected of physicians or dentists at the time of licensing [http://www.mft-online.de/files/nklm_nklz_information_20130419_kurz.pdf, last verified on 14 December 2014]. A national consensus regarding the catalogue’s content was expected to be reached by May 2015.

**Figure 1:**
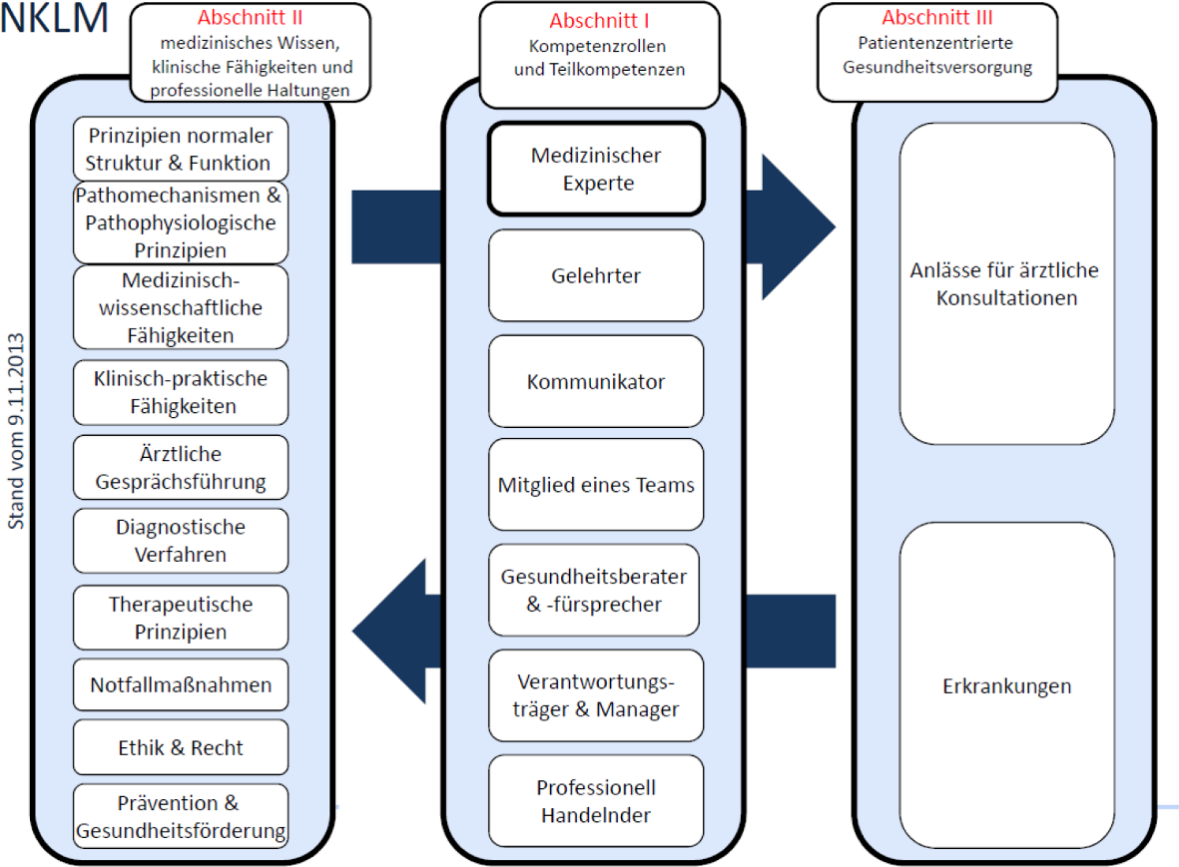
Structure of the national competence-based learning objectives for undergraduate medical education in Germany, NKLM [http://www.mft-online.de/files/nklm_nklz_information_20130419_kurz.pdf, last verified on 14 December 2014].

The national catalogue of competency-based learning objectives for undergraduate medical education (NKLM) is divided into three sections, with the first section dealing with competency roles and competencies; the second section addressing medical knowledge, clinical skills and professional decision-making and the third one covering patient-centred healthcare (see Figure 1 (Fig. 1)). The NKLM contains overarching competencies as roles, which are based on the CanMEDS model [http://www.royalcollege.ca/portal/page/portal/rc/canmeds/framework, last verified on 14 December 2014].

The goals of the NKLM include not only the integration of learning objectives in teaching but also the assessments conducted in medical education. With the implementation of the NKLM in medical education, there are new requirements to expand on established forms of assessment and to develop innovative testing formats to measure competencies.

Within the scope of the joint project “Medical Education Research –* Lehrforschung im Netz BW*” (MERLIN) [http://www.merlin-bw.de/, last verified on 14 December 2014], promoted by the German Federal Ministry of Education and Research as part of the Quality Pact for Teaching [http://www.bmbf.de/de/15375.php, last verified on 14 December 2014], the call to develop a competency-based progress test was answered by the University of Heidelberg’s Center of Excellence for Assessment in Medicine and was combined with the involvement of students to design the test to develop a competency-based progress test suitable for providing longitudinal feedback.

This requires a step-by-step approach. The initial starting point for the development of a competency-based progress test and the focus of this study are the knowledge components that are present in each competency (see the steps to knowledge according to North [27] or Miller’s Pyramid [28]). These components of knowledge, as a theoretical aspect of competence, can be assessed using MCQs. Even if competencies are to be assessed eventually, to the maximum extent possible, as a whole on a higher level than just in relation to the related knowledge components, this pilot study represents the first step in the direction of a competency-based progress test.

A comparison of established progress tests shows that many blueprints have been used in which a focus on competency is present in sections but does not represent a design criterion of the blueprint itself. The test questions (generally type A MCQs) are developed on the basis of one- or two-dimensional blueprints using organ systems, disciplines or tasks [http://ptm.charite.de/, http://www.nbme.org/Students/sas/ifom.html and http://www.ivtg.nl/en, all of which were last verified on 14 December 2014]. However, developing a blueprint for a competency-based progress test requires mapping out the performance requirements in the medical degree programme and competency-based education and applying these as the design principle.

In this study, the design of a competency-based blueprint is explained and attention is provided to the following issues.

1. To what extent can students be included in the development of a progress test?
2. Is it possible to reliably incorporate knowledge components of competencies in a progress test that consists of MCQs?

## Methods

### Blueprint development for a competency-based progress test

Both the current German medical licensing regulations (ÄAppO) [http://www.gesetze-im-internet.de/_appro_2002/BJNR240500002.html, last verified on 14 December 2014] and the NKLM were drawn upon to design the blueprint. The goal was to incorporate the 55 subjects listed in the ÄAppO and the 17 relevant areas of the NKLM (sections I and II) in a two-dimensional blueprint for a written assessment. To make the blueprint feasible for compiling an examination, the number of cells must be limited, for which the subjects and the NKLM areas were put into groups. As part of the ItemManagementSystem (IMS) conference in January 2013, three inter-university and interdisciplinary expert groups were formed and, as a first step, each given the task of independently placing the NKLM areas into clusters of competency areas. In a second step, the expert groups were assigned the task of building clusters of subjects based on those listed in the ÄAppO in such a way that each cluster contained pre-clinical and clinical subjects in as equal a measure as possible.

When generating MCQs, the blueprint guarantees that each of the questions will reflect the integration of a subject group and a competency area.

#### Recruiting students

Students were recruited by contacting German medical schools requesting them to cooperate and forward the information to interested students. Initially, 31 students from seven medical schools in Germany (Düsseldorf, Freiburg, Heidelberg, Magdeburg, Marburg, Tuebingen and Witten-Herdecke) participated in the development of the progress test. A core team emerged comprising 17 students in their third to ninth semesters of study from five universities (Freiburg, Heidelberg, Magdeburg, Marburg and Witten-Herdecke). All of the participating students were offered work-study contracts as a means of ensuring long-term cooperation, and a majority of them accepted this offer.

#### Training students

The students were extensively trained in five 2-day workshops, which occurred between February and October 2013. The following topics were covered:

- competency-based assessment
- CanMeds role models
- concept of the progress test
- blueprint of the competency-based progress test
- formative and summative assessments
- quality criteria for MCQs
- group review of MCQs
- working with the IMS

During the working phases, the students were responsible for using the blueprint to draft appropriate MCQs regarding the subject groups and competency areas. This was accomplished partly during the workshops and partly in self-organized, small groups using a separate platform of the IMS dedicated solely to this progress test [https://www.ucan-assess.org/cms/de/tools/item-and-exam-management/, last verified on 14 December 2014]. This included the following topics:

- MCQ generation
- group review
- revision of the MCQs
- re-review

Group reviews together with the trainers occurred during the workshops as a quality assurance measure.

The process of having students write MCQs for the formative competency-based progress test was presented by a team of committed students at the 2013 annual convention of the *Gesellschaft für Medizinische Ausbildung* in Graz [29].

#### MCQ pool 

Exclusively type A MCQs were used in the pool of questions for piloting the formative competency-based progress test. The entire MCQ pool was then reviewed by university lecturers with regard to form and content.

#### Piloting the formative competency-based progress test

Pilot administration of the formative competency-based progress test occurred at the end of November 2013 at eight medical schools. This included the medical schools that participated in the MERLIN project in Baden-Württemberg (Freiburg, Heidelberg, Mannheim, Tuebingen and Ulm) and three other medical schools at the Universities of Magdeburg, Marburg and Witten-Herdecke. Announcement of the progress test, student registration and on-site administration were independently undertaken by each medical school. Leadership laid with the University of Heidelberg’s Center of Excellence for Assessment in Medicine.

The time allotted for 144 MCQs was 3 h, providing the students the option of finishing the progress test earlier.

Before starting the test, students were provided standardized instructions – analogous to a “don’t know”-option – not to guess the answers of questions they did not know, so as to minimize the effect of guessing [3], [30].

#### Quality of the progress test and results based on academic year

The reliability of the entire test and those for the sections on subject groups and competency areas, as defined by the two blueprint axes, were measured by calculating the internal consistency (Cronbach’s α). To allow comparison of the reliabilities for the scales consisting of different numbers of questions, the reliability standardized for test length 1 was also calculated using the Spearman–Brown formula.

The percentages of correctly answered and unanswered questions (don’t know) were determined depending on the academic level of the participating students.

Discriminant validity was analysed for the competency areas. Following principal component analysis of the data [31], discriminatory analysis was conducted on the questions in the individual competency areas (a detailed description of this procedure can be found elsewhere [32]).

#### Representativeness

Regarding the representativeness, it must be noted that the participant sample cannot be viewed as representative due to the voluntary nature of participation and the different recruiting methods. Whether or not the data are subject to a selection bias as a result (e.g. if the more successful or less successful students participated) cannot be determined within the context of this pilot study because no comparative data could be gathered on participants and non-participants.

#### Feedback on the formative competency-based progress test

A procedure for giving feedback was designed for the formative competency-based progress test to tell students where they stand in terms of performance and to give schools an overview of the students’ performance in each academic year:

1. The participating students received specific feedback regarding their performance in the medical programme compared with cohorts at the same semester level at their school not only in the form of an overall summary but also differentiated according to subject groups and competency areas. (An example of this feedback can be viewed online [https://www.ucan-assess.org/cms/networks/student-progress-test/, last verified on 14 December 2014]).
2. The universities received information on the cohorts according to the academic year in summary form and differentiated according to subject groups and competency areas.

A comparison of the participating medical schools was not undertaken due to differences in curricular designs and the assumption that the participants were not representative.

#### Evaluation

The evaluation of the formative competency-based progress test was conducted by Freiburg University’s Competence Center for Evaluation of Teaching in Medicine, one of the MERLIN project partners. Immediately after taking the progress test, all of the students responded to a brief survey of 11 questions about the progress test.

## Results

### Blueprint of the competency-based progress test

**Figure 2:**
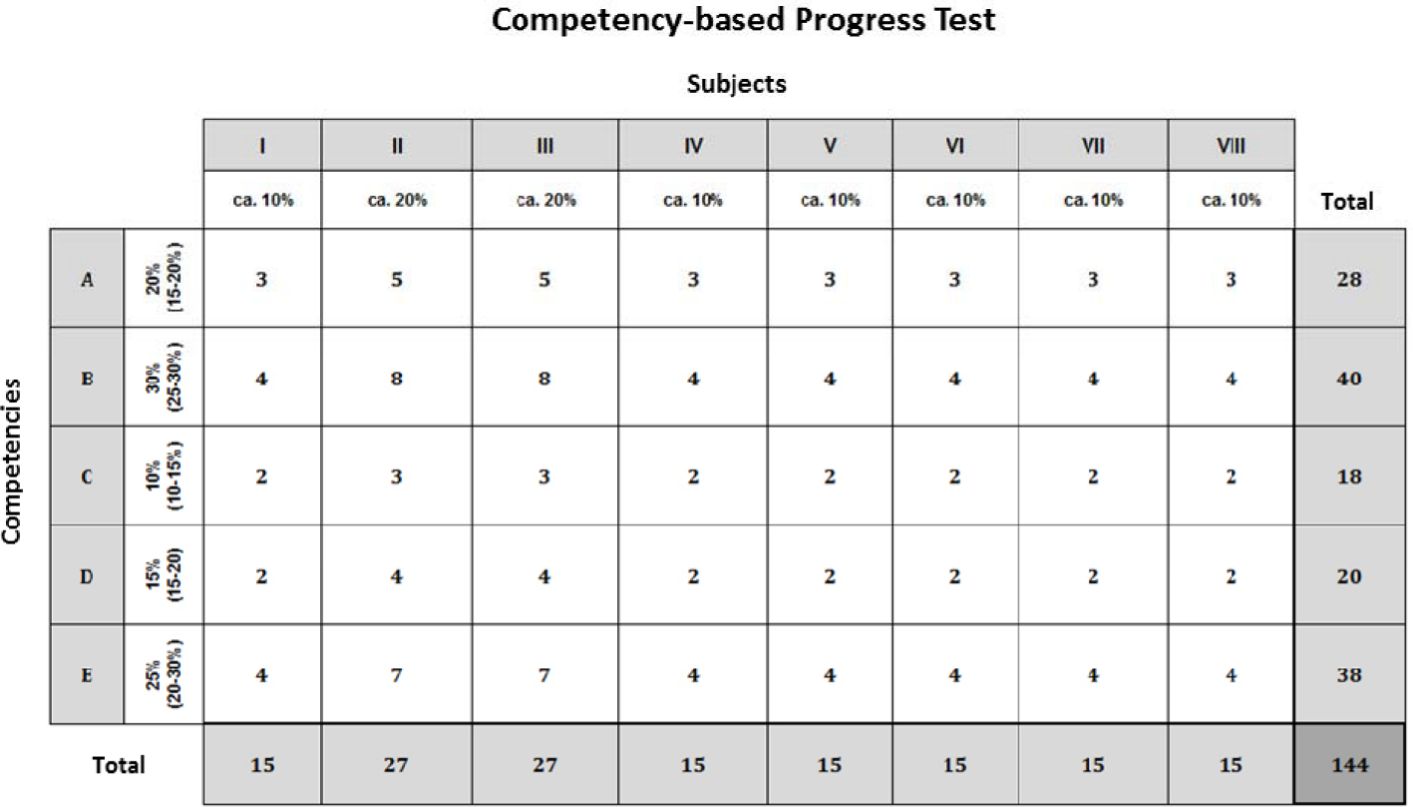
Blueprint of the formative competency-based progress test: weighting of the subject groups and competency areas and the resulting item numbers.

A two-dimensional blueprint emerged from the consensus reached by the expert groups. The 17 relevant areas in the NKLM were clustered into five weighted competency areas and the 55 disciplines defined by the ÄAppO into eight weighted subject groups (see Table 1 (Tab. 1)). The blueprint contains 144 items to allow for both the weighting and an appropriate amount of time to take the test (see Figure 2 (Fig. 2)).

#### Student generation of MCQs

For the pilot study, the students generated 207 reviewed questions that covered 118 of the 144 blueprint MCQs. Examples of MCQs integrating a subject group and a competency area as foreseen by the blueprint are presented in table 2 (Tab. 2).

**Figure 3:**
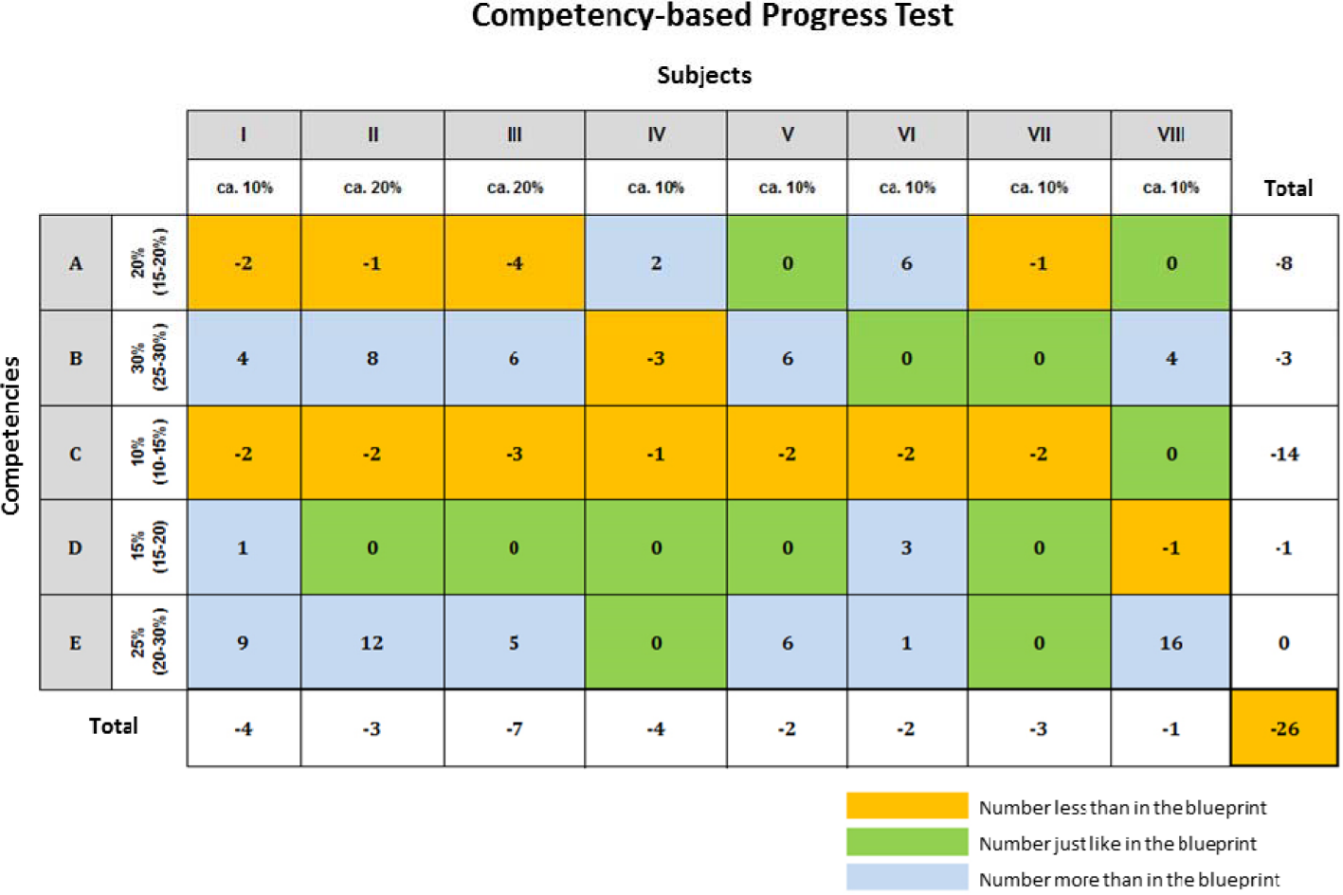
Fulfilment of the blueprint of the formative competency-based progress test by student-generated MCQs. The number of missing MCQs is summed.

Figure 3 (Fig. 3) shows that very few MCQs were generated for Competency Area C (Scientific Skills) and also in part for Competency Area A (Communication Skills). To cover the entire blueprint, the 26 additionally required questions were provided by instructors.

#### Pilot study: participating students

A total of 469 students participated in the pilot study conducted at eight medical schools at the end of November 2013, during which the formative competency-based progress test was administered. The number of students per school or per academic year was subject to great variance (see Table 3 (Tab. 3)).

#### Results of the MCQs

##### Quality of the MCQs

All of the MCQs were subjected to a post-review and evaluated in terms of statistical values. Of the total 144 MCQs, which also included the 26 questions written by instructors, 31 questions were checked due to conspicuous statistical values with respect to the level of difficulty (P≤0.40 and P≥0.85) and the corrected discriminating power (r‘≤0.20), wherein the corrected discriminating power is the product-moment correlation of the points for the question with the sum of the points of all other questions. After another expert review of the statistically conspicuous MCQs, an additional response was counted as correct for five of these MCQs. Of these five questions, three were written by students and two by instructors. Four student-generated MCQs were excluded from the evaluation leaving a total of 140 MCQs in the analysis.

##### Test values

A mean of 69.38 points out of 140 possible points was scored (SD=23.69). The difficulty of the overall test was thus P=0.496.

##### Reliability

The reliability of the entire progress test (n=140) was α=0.954 (Cronbach’s α). The overall reliability, the reliability differentiated according to subject groups and competency areas and the reliability according to the academic year are presented in Table 4 (Tab. 4).

##### Don’t know-option

All unanswered MCQs were interpreted as “don’t know” responses due to the standardized instructions provided to all participating students at the beginning of the progress test.

##### Scores

**Figure 4:**
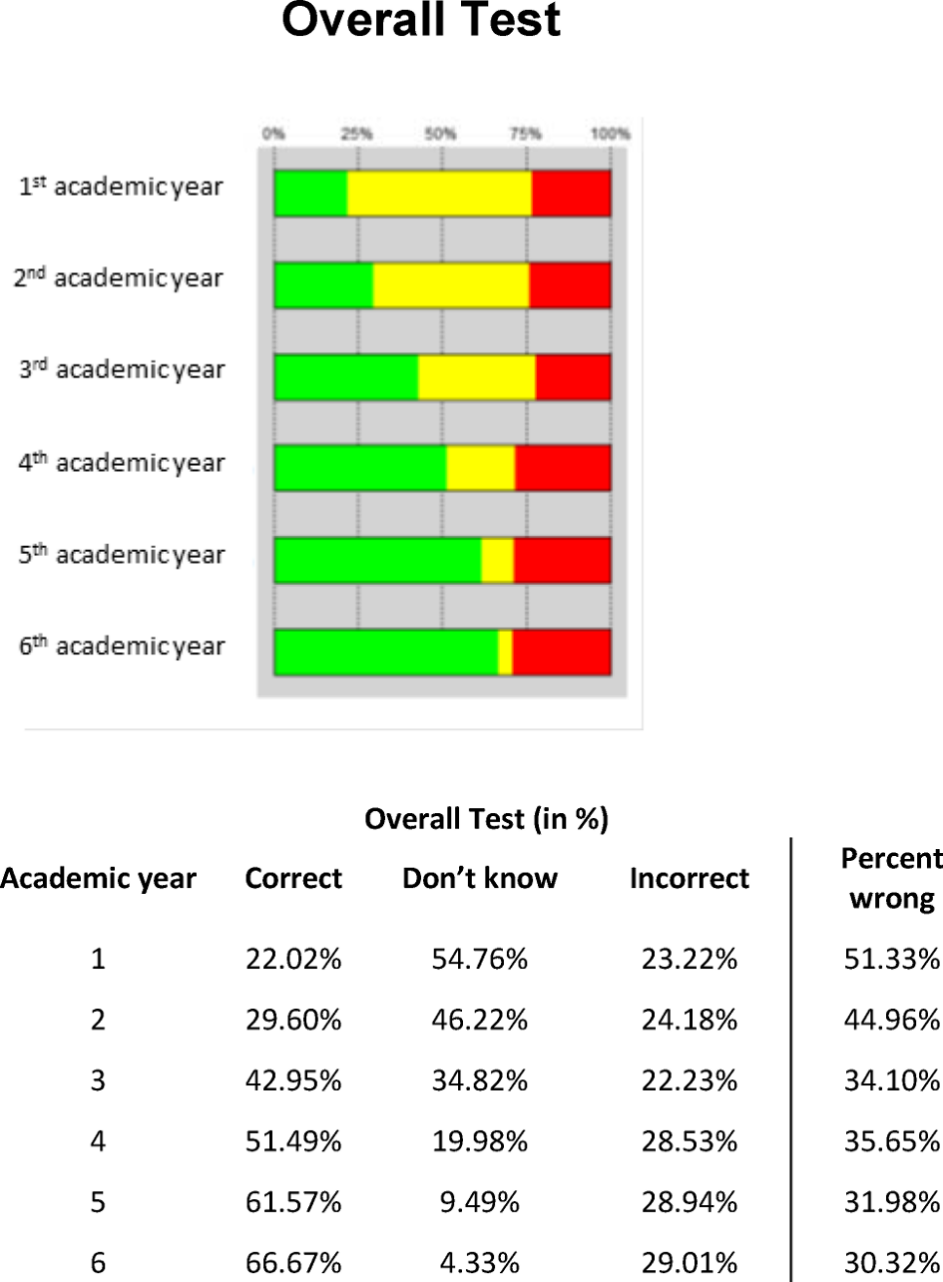
Results of the progress test at all eight schools (n=469): Overall test; correct (green), don’t know (yellow), incorrect (red) and percent wrong (incorrect / (correct + incorrect)).

**Figure 5:**
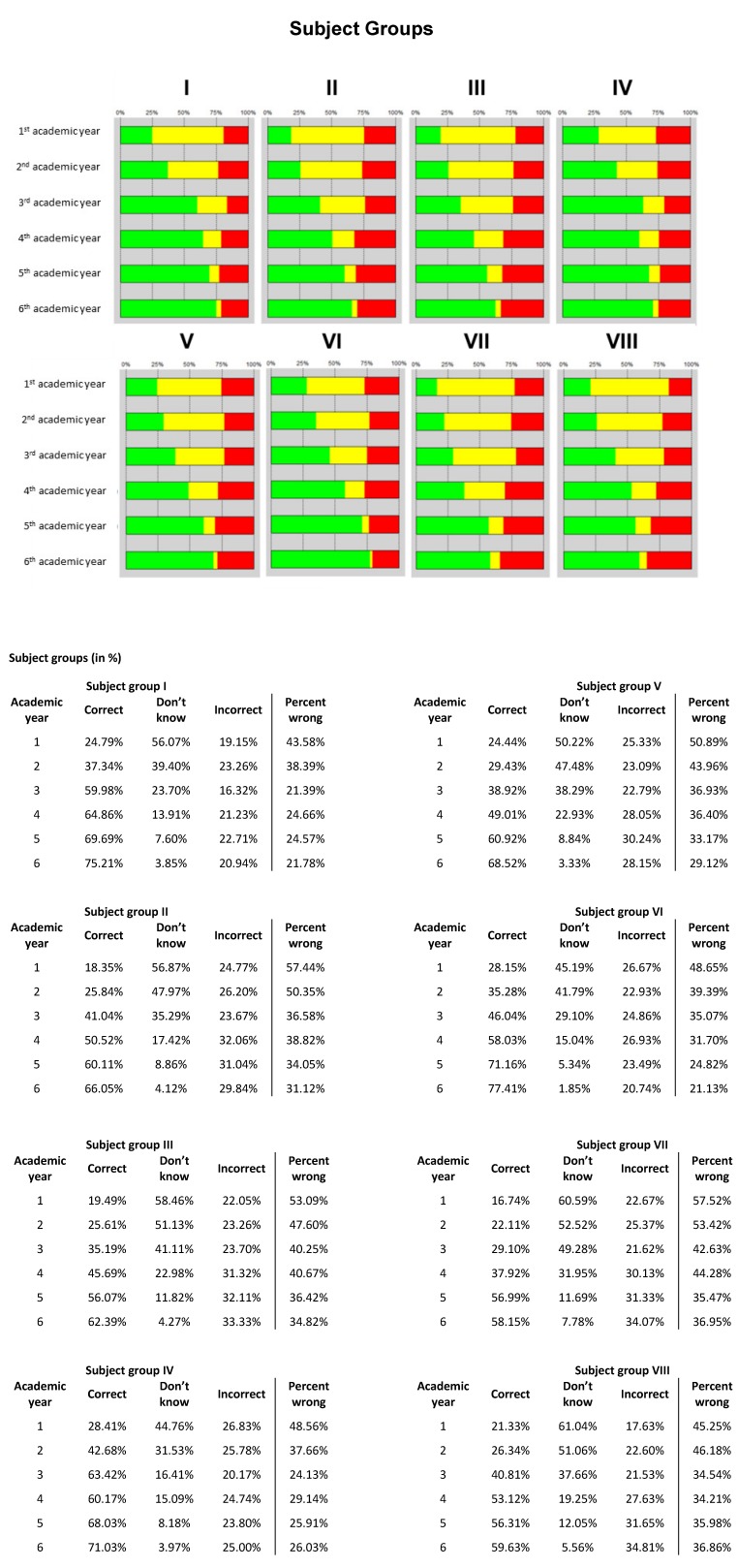
Results of the progress test at all eight schools (n=469): Subject groups; correct (green), don’t know (yellow), incorrect (red) and percent wrong (incorrect / (correct + incorrect)).

**Figure 6:**
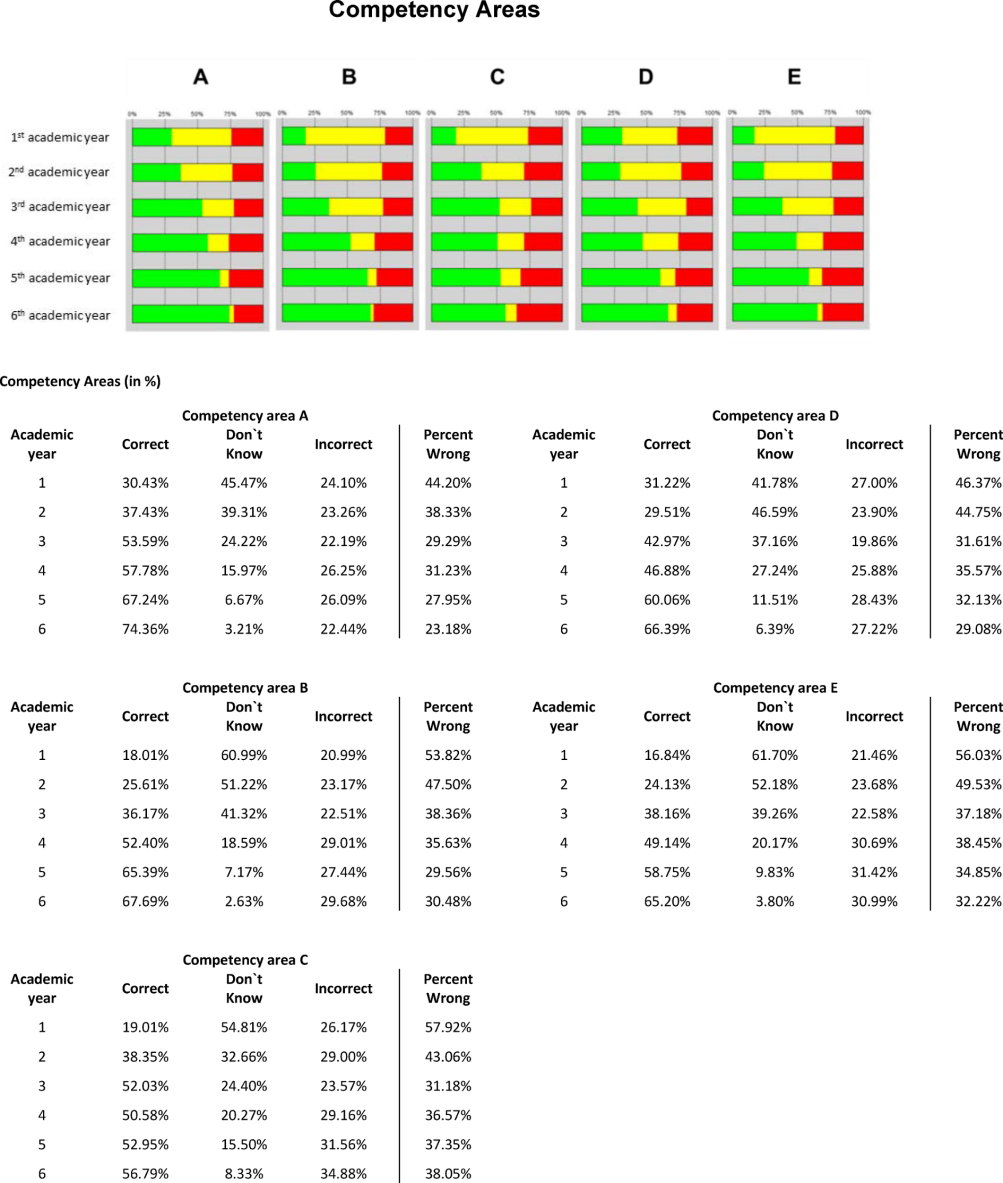
Results of the progress test at all eight schools (n=469): Competency areas; correct (green), don’t know (yellow), incorrect (red) and percent wrong (incorrect / (correct + incorrect)).

The number of correct answers increased steadily with advancing semester level (see Figure 4 (Fig. 4)). This is demonstrated by the overall analysis of the right answers (22.02% in the first academic year to 66.67% in the sixth academic year) and also by the differentiated analysis of the subject groups and competency areas (see Figure 5 (Fig. 5) and 6 (Fig. 6)). Similarly, the number of ‘don’t know’ responses decreased with advancing semester level. Although the number of wrong answers slightly increased absolutely with advancing semester level, it declined in relation to the number of questions answered as the semester level progressed (percentage of wrong answers).

#### Evaluation

The total number of students who participated in the evaluation was 463, of which 284 (61.3%) were females, 162 (35.0%) were males and 17 without an indication of their gender. The mean age of the participating students was 24.56 years (SD=3.30). The results of the 11 items are given in Table 5 (Tab. 5).

## Discussion

This multi-centre pilot study demonstrates how the formative progress test format was developed and implemented as a competency-based test. Clustering the 55 disciplines from the ÄAppO and the 17 work packages of the NKLM according to the content in a two-dimensional blueprint with eight subject groups and five competency areas has proved to be a viable instrument for developing MCQs for the competency-based progress test.

This study is also a positive example of student involvement in medical education. Involving students in the development of the progress test and writing the MCQs, as well as the conduction of the pilot study at eight medical schools, was both feasible and successful.

However, there were also limitations in designing the pilot study with respect to the drafting of MCQs by the students. As Figure 3 (Fig. 3) shows, the students were not able to come up with enough questions particularly for the Competency Area C (Scientific Skills) and in part for Competency Area A (Communication Skills). This may suggest that, when designing the pilot study, the students were not able to recognize these competency areas from their own studies or to think of a sufficient number of good scenarios, which implies that more intensive training in these competency areas is required. Comparing these two areas with the number of questions written for Competency Areas B (Practical Clinical Skills) and E (Theoretical Clinical Skills) shows that many more MCQs were generated for these areas, since their combination with the subject groups allows for MCQs of a more ‘classic’ nature.

The drafting of MCQs by trained students based on the blueprint led to MCQs of a very high quality, which is confirmed by the low number of corrected (5) and excluded (4) MCQs out of the 144 questions in the post-review. However, it must be noted that the difficulty of the student-generated MCQs must be considered too high, with P=0.496 in relation to all academic levels, and P=0.667 for the sixth academic year in the pilot study.

Overall, the formative competency-based progress test demonstrates a high reliability. Similarly, the reliabilities are sufficiently high for the subject groups, competency areas and among the academic levels.

A significant result is that the formative competency-based progress test shows growth of knowledge in the cross-sectional view of all academic years [33], [34]. This applies not only to the overall consideration of the progress test but also with regard to the subject groups and competency areas. The number of correct answers increased steadily over time, while the number of “don’t know” responses and the percentage of wrong answers diminished steadily in relation to the answered MCQs. The latter finding might indicate that students are able to recognize answerable MCQs with more confidence as they progress to more advanced semester levels. 

This study shows that the theoretical knowledge forming the basis of competence (in terms of the steps to competence based on North [27] or Miller’s pyramid of competence [28]) can be reflected in a competency-based progress test with MCQs drafted by students. However, competence is only captured as an aspect of knowledge in this competency-based progress test.

Future studies should explore whether competency-based test questions drafted by students can be successfully applied to other testing formats that can assess skills at a higher level, as is possible with key feature questions and situational judgement questions, to evaluate professional decision-making [35], [36] or OSCEs.

Aspects such as the focus on competency and the related blueprint must also be discussed with regard to further development of the competency-based progress test. It has already been possible to demonstrate using the discriminant validity analysis of the five competency clusters that these clusters are indeed reflected in the empirical results and that differentiation according to these groups makes sense when scoring and providing feedback to students and medical schools [32] (see Table 6 (Tab. 6)).

Evaluation of the pilot test at eight medical schools shows that students were highly motivated to take the progress test and become familiar with this exam format (see Table 5 (Tab. 5)). The students expressed a desire to have the progress test as a permanent part of the study programme and to receive helpful and altogether more feedback on their performance in the medical programme (items 1, 2, 7, 8 and 10). Since the progress test was offered as a formative test, these high values were to be expected. The questions about the administration of the progress test (items 4 and 5) were answered positively overall, whereby certain concessions must be made with respect to informing students in advance about the concept of progress testing (item 3). Regarding the design of the progress test, students cast a clear vote in favour of having it administered as a formative test (item 9). The majority of students preferred an exam frequency of once a year (item 11). However, only in a limited sense did the students perceive it as a test with a learning effect (item 6). If this question is considered in connection with the desire for more helpful feedback on the proficiency levels during the medical programme (item 7), it indicates the future direction for developing feedback for students, even though no direct learning effect can be expected based on taking the progress test alone. Nonetheless, the feedback offers learning opportunities in two ways. Through detailed feedback on individual responses to the separate questions, the knowledge tested by the progress test can be subsequently learned. Of more interest is the feedback on the subject groups and competency areas, which can be used by the participating students to define the focus of their studies according to specific strengths and weaknesses.

## Outlook

Based on the results of this multi-centre pilot study using the formative competency-based progress test with student-generated MCQs, further development and use of this exam format is aimed for with the active inclusion of students in the process. The focus of continued development will be on the feedback for participating students, the type of test administration (e.g. online test) and the expansion to include other questions and exam formats (e.g. key feature questions, situational judgement questions and OSCE) that are able to represent the basis of competency at a higher level than is possible with MCQs.

## Competing interests

The authores declare that they have no competing interests.

## Figures and Tables

**Table 1:**
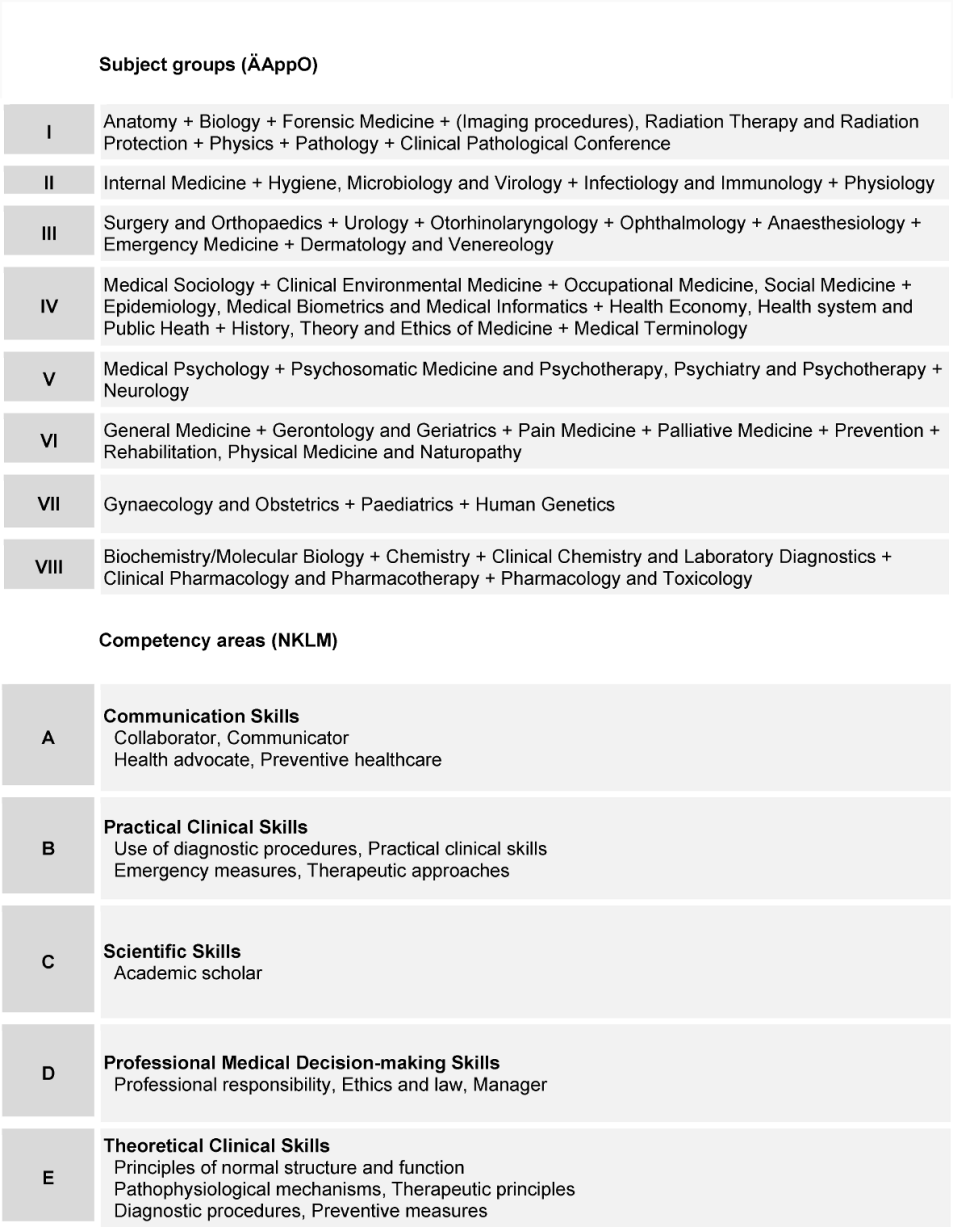
Blueprint of the formative competency-based progress test: subject group and competency area clusters.

**Table 2:**
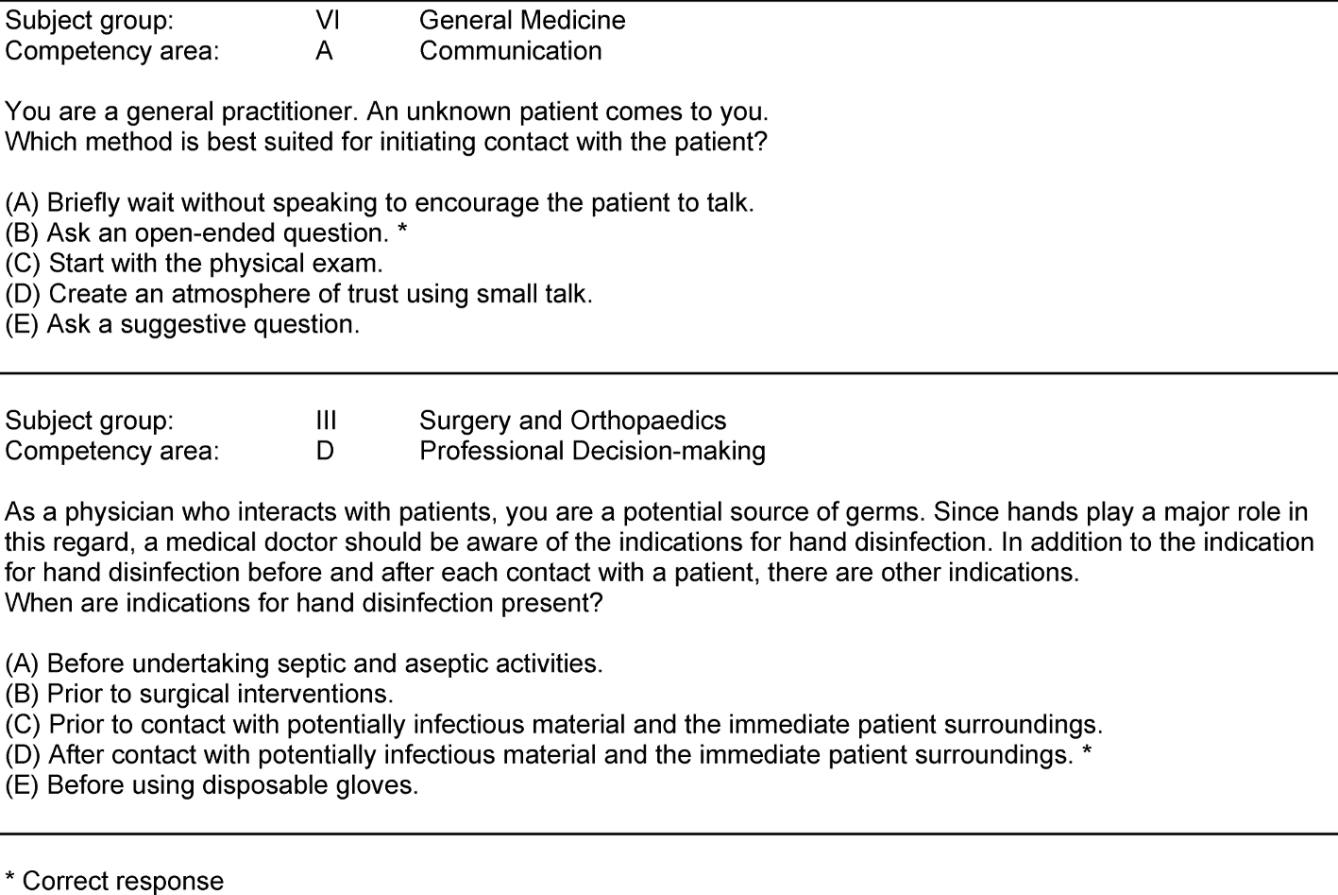
Examples of student-generated MCQs integrating subject group and competency area [https://www.ucan-assess.org/cms/networks/student-progress-test/].

**Table 3:**
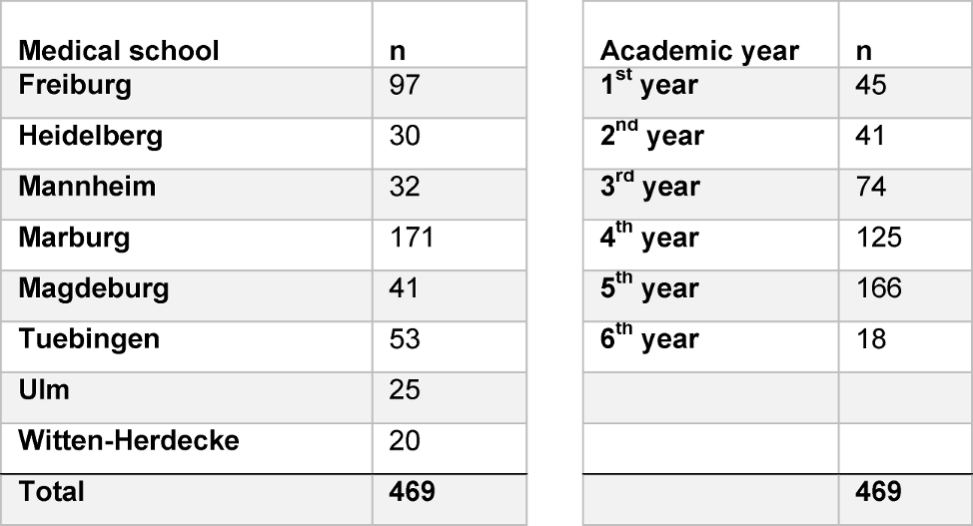
Number of students participating in the pilot study conducted in November 2013 at eight medical schools.

**Table 4:**
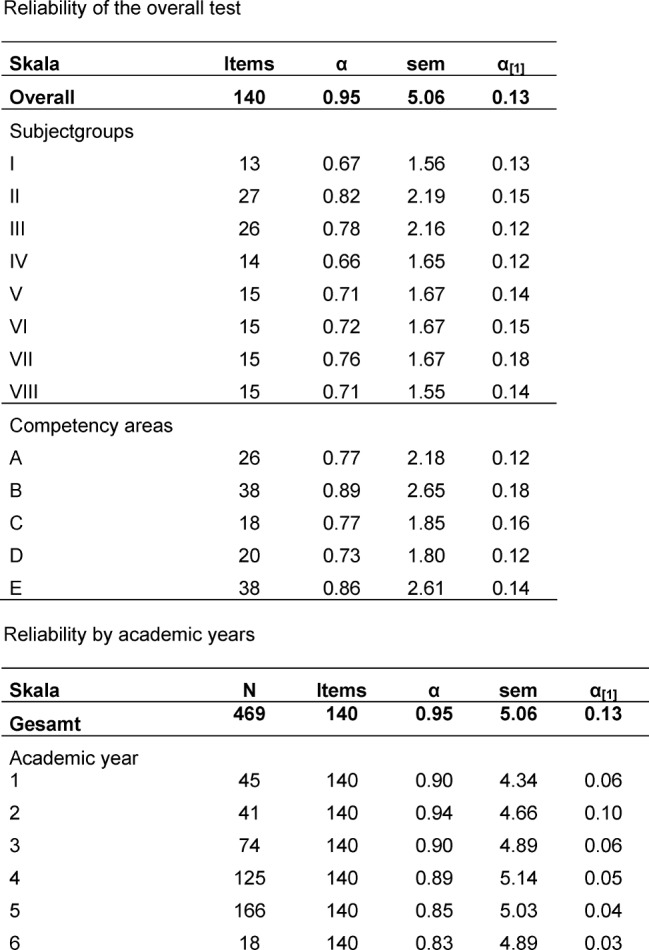
Reliability of the entire test and of the subject groups, competency areas and academic years: internal consistency (Cronbach’s α), standard error of measurement for the scale (sem), standardized reliability for test length 1 according to the Spearman–Brown formula (α_[1]_).

**Table 5:**
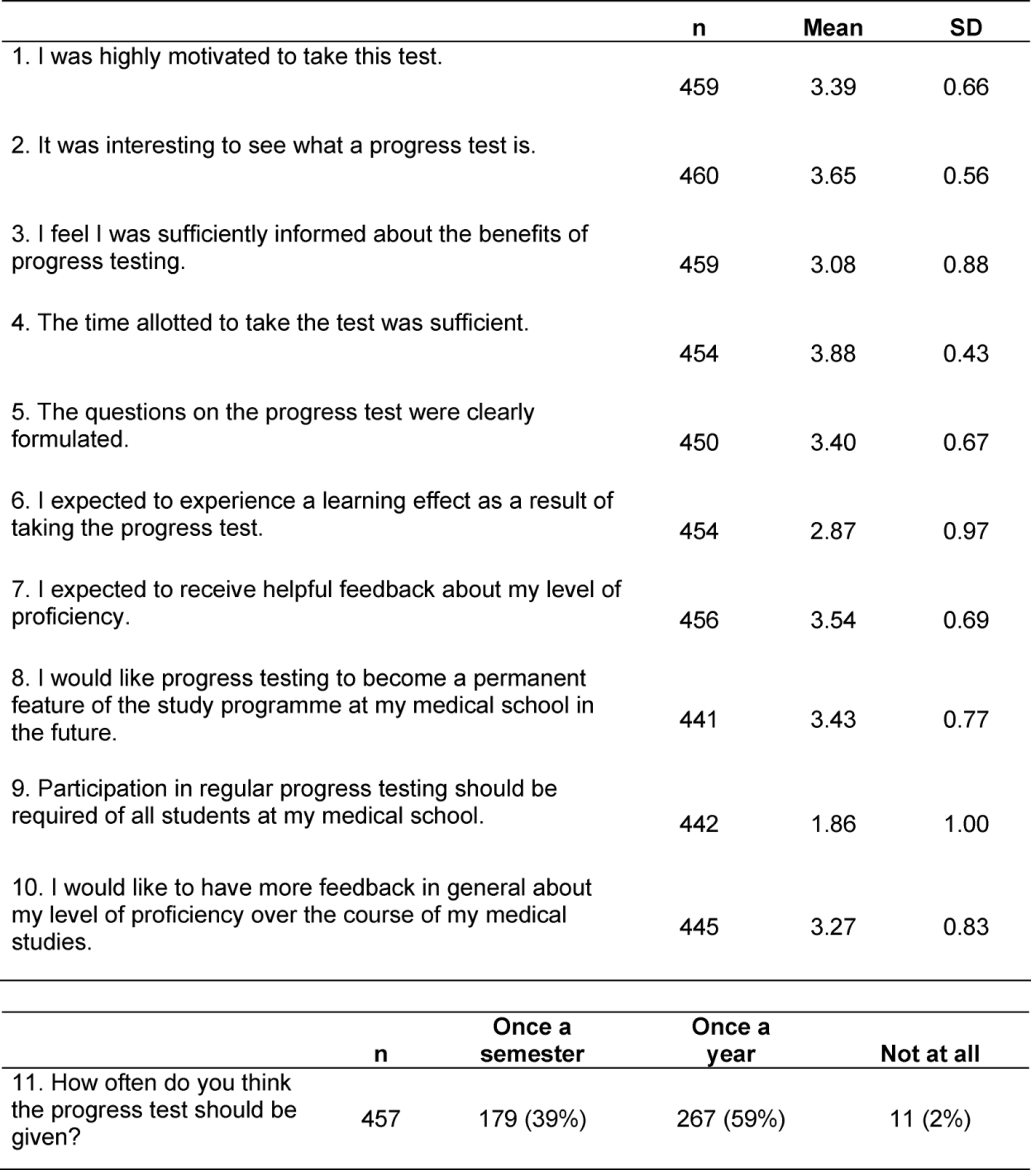
Results of the progress test evaluation at eight schools (n=463). The survey was conducted using a 4-point scale (1=strongly disagree, 2=disagree, 3=agree and 4=strongly agree) with the additional option “not applicable”.

**Table 6:**
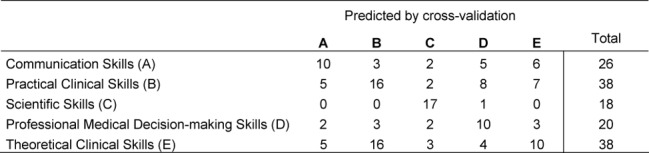
Discriminant validity analysis of the questions in the competency areas based on the four principal components defined by principal component analysis of the data. Results of the cross-validation according to the leave-one-out method.
